# Fine-Scale Mapping of Natural Variation in Fly Fecundity Identifies Neuronal Domain of Expression and Function of an Aquaporin

**DOI:** 10.1371/journal.pgen.1002631

**Published:** 2012-04-05

**Authors:** Alan O. Bergland, Hyo-seok Chae, Young-Joon Kim, Marc Tatar

**Affiliations:** 1 Department of Ecology and Evolutionary Biology, Brown University, Providence, Rhode Island, United States of America; 2 Department of Biology, Stanford University, Stanford, California, United States of America; 3 School of Life Sciences, Gwangju Institute of Science and Technology (GIST), Gwangju, Republic of Korea; University of Southern California, United States of America

## Abstract

To gain insight into the molecular genetic basis of standing variation in fitness related traits, we identify a novel factor that regulates the molecular and physiological basis of natural variation in female *Drosophila melanogaster* fecundity. Genetic variation in female fecundity in flies derived from a wild orchard population is heritable and largely independent of other measured life history traits. We map a portion of this variation to a single QTL and then use deficiency mapping to further refine this QTL to 5 candidate genes. Ubiquitous expression of RNAi against only one of these genes, an aquaporin encoded by *Drip*, reduces fecundity. Within our mapping population *Drip* mRNA level in the head, but not other tissues, is positively correlated with fecundity. We localize *Drip* expression to a small population of corazonin producing neurons located in the dorsolateral posterior compartments of the protocerebrum. Expression of *Drip*–RNAi using both the pan-neuronal *ELAV*-*Gal4* and the *Crz-Gal4* drivers reduces fecundity. Low-fecundity RILs have decreased *Crz* expression and increased expression of *pale*, the enzyme encoding the rate-limiting step in the production of dopamine, a modulator of insect life histories. Taken together these data suggest that natural variation in *Drip* expression in the corazonin producing neurons contributes to standing variation in fitness by altering the concentration of two neurohormones.

## Author Summary

A major goal of modern evolutionary biology is to elucidate the genetic basis of standing genetic variation underlying fitness traits. This goal is important for a comprehensive picture of the evolutionary process, because it allows us to understand the mode of natural selection on fitness traits and identify the molecular and physiological processes that affect fitness traits. Here, we describe our work to identify the molecular genetic and physiological basis for natural variation in a core life history trait, fecundity, of *Drosophila melanogaster*. Using a variety of mapping techniques, we show that differential expression of the aquaporin *Drip* in nervous tissue affects natural variation in female fecundity. We further go on to describe a novel domain of expression of *Drip* in neurons that produce the insect stress hormone corazonin and demonstrate that differential expression of *Drip* in these neurons affects female fecundity putatively through modulating the concentration of corazonin and dopamine. This surprising and novel observation highlights the benefit of exploiting natural genetic variation to identify the molecular processes underlying phenotypic traits.

## Introduction

The life history of an organism—its reproductive schedule and lifespan—are fundamental characteristics intrinsically related to its evolutionary fitness. Due to the close relationship of life history with fitness, theory predicts life history traits should be subject to strong natural selection [1], [2]. However, the exact mode of action of natural selection on these traits is often complex and context dependent [3], [4]. Environmental heterogeneity, overdominance, frequency dependent selection or life history tradeoffs may thus maintain genetic variation in fitness traits [4]–[7]. Due to the complex behavior of natural selection on life history traits, there has been great interest to quantify their magnitude of genetic variation and to map natural alleles underlying this variation.

These efforts have mapped natural variation in life history traits to specific loci in a variety of organisms [8]. In *Arabidopsis thaliana* genetic variation in reproductive timing in response to vernalization has been mapped to polymorphisms in FLOWERING LOCUS C [9], [10] and FRIGIDA [11]. Genetic variation in population growth rate and dispersal in the Glanville fritillary butterfly, *Melitaea cinxia*, is associated with polymorphism in *phosphoglucose isomerase* and *succinate dehydrogenase* [12]. In the fruit fly, *Drosophila melanogaster*, genetic variation in reproductive diapause has been mapped to components of the insulin signaling [13], circadian clock [14], and neuronal development pathways [15]. For *D. melanogaster* in particular, extensive work has examined natural variation in age specific survival, a core life history component. Using traditional quantitative trait locus (QTL) mapping techniques, dozens of positional loci affecting lifespan have been identified [16]–[26]. Several of these QTL have been localized to specific genes including *Dox-A2, tup, ms(2)35Ci, stc, Lim3, Ddc*, and *catsup*; [18], [27]–[31]. A complimentary approach has described natural polymorphisms for lifespan in pro-longevity genes such as the G-coupled receptor *methuselah* [32] [33] that were previously identified through molecular techniques.

While considerable progress has been made uncovering the genetic basis for natural variation in lifespan, relatively little is known about natural variation in reproductive output. Natural genetic variation in only two genes has been associated with variation in fecundity (*mth* and *InR*; [33], [34]) although several studies have identified positional QTL for age specific female fecundity [35], male mating success [36] and ovariole number [37], [38] without resolving a molecular or specific genetic determinant. To advance this issue we identify novel genetic loci affecting fecundity in a natural population of *D. melanogaster*. We document segregating genetic variation in fecundity and identify a QTL underlying this variation. We localize the polymorphism in this QTL to a region containing 5 genes including the aquaporin, Drip. *Drip* expression in head tissue is variable in our mapping population as is two genes putatively downstream of Drip, *Crz* and *pale*. Drip has been previously described to function in malphigian tubules where it serves to regulate water balance [39] in an ecdysone dependent fashion [40]. Here we show that *Drip* is expressed in a small population of corazonin producing neurons in the fly brain and *Drip* expression in these neurons modulates fecundity. This work localizes a new polymorphic locus that effects a core life history trait and identifies a novel function and domain of expression for *Drip*.

## Results/Discussion

To understand the genetic basis of phenotypic variation in female fecundity, we studied a set of 12 recombinant inbred lines (RILs) derived from an orchard population in Winters, CA. These RILs were chosen to maximize genetic variance in ovariole number and thorax length, two morphological determinants of fecundity [38]. RIL larvae were reared in the lab on diets of several yeast concentrations to identify nutrient dependent effects on female fecundity, egg-to-adult development time, ovariole number and thorax length. Significant genetic and genotype-by-environment variation is seen for each of these life history traits (Tables S1, S2, S3, S4). Measured as phenotypic variation among individuals, larger females have more ovarioles, develop quicker and lay more eggs, and standard and partial correlations between these traits are generally independent of larval rearing environment (Table S5, Figure S1A–S1F). We analyzed standard and partial correlations among line means to estimate the genetic contributions to these phenotypic patterns. Genetically, ovariole number and thorax length are positively correlated, and again independent of larval rearing environment (Table S5; *cf* [38]). On the other hand, total fecundity is only correlated with ovariole number when larvae are reared in the high larval yeast environment but this correlation does not appear when examining partial correlations (Table S5, Figure S1A–S1F). The discrepancy between phenotypic and genetic correlations for these traits suggests that the primary cause of the observed phenotypic correlations between fecundity and other measured life history traits derives from micro-environmental variation in food concentration within vials or asymmetric competition within rearing vials. Taken together these results suggest that genetic variation for fecundity is largely independent of other measured life history traits in this mapping population.

**Figure 1:**
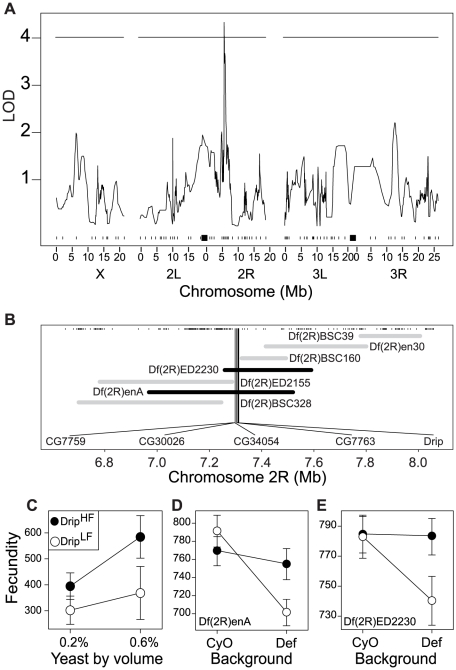
QTL and fine-scale mapping of female fecundity localizes to 5 positional candidate genes. (A) QTL scan results for fecundity. The *x*-axis represents position along the three major *D. melanogaster* chromosomes in megabases (Mb). Tick marks represent location of polymorphic markers used for QTL mapping and black squares represent the approximate location of the centromeres. The *y*-axis represents the strength of association between a particular region and fecundity. The horizontal line represents the 95% permutation threshold. (B) Deficiency map of the QTL region on 2R. The *x-axis* represents position along chromosome 2R in megabases (Mb). Tick marks represent location of known genes and the horizontal bars represent the location (either molecularly defined or approximate) of deficiency break points. Grey bars represent deficiencies that complemented the RIL alleles, black bars represent deficiencies that failed to complement the RIL alleles. The five named genes are those genes identified by quantitative complementation as candidates genes affecting fecundity. (C) Estimated effect of the two RIL alleles at the QTL identified on chromosome 2R. Black and white circles represent high and low fecundity alleles, respectively. The *x*-axis represents larval rearing condition. The *y*-axis represents estimated fecundity. Error bars represent 95% CI calculated from amongst line variance. (D–E) Results from quantitative complementation tests with the two deficiencies that failed to complement the two alleles in the mapping population. The *x*-axis of each inset represents the tester chromosome (either “wild-type”- CyO, or deficiency – Def). Black vs. white circles represent the high and low fecundity RIL alleles, respectively. The *y*-axis of each inset represents estimated fecundity (see Materials and methods for more details). Error bars represent 95% CI based on non-parametric bootstrap resampling (5000 replicates), conditional on fly.

Given the genetic contribution to phenotypic variance in these traits within the population, we sought to identify chromosomal regions associated with these traits and their response to the environment. Multiple imputation QTL mapping [41] with ∼100 SNP markers spread along the three major *D. melanogaster* chromosomes [38] did not identify any significant QTL affecting ovariole number, thorax length or development time but did locate a single QTL affecting fecundity in chromosome 2R between bands 47D–E to 48E (Figure 1A) explaining ∼35% of the observed genetic variance. The effect of this QTL on fecundity is greater in flies reared in 0.6% yeast than flies reared in 0.2% yeast (Figure 1C). As expected from the small number of lines used for this analysis, we detect only a limited number of QTL; there is likely to be considerable unmapped genetic variation for these traits in our population and in the wild population from which these lines were derived.

The chromosome 2R genomic region affecting fecundity contains approximately 150 genes within the QTL bound by a 3-LOD interval. We used quantitative complementation with deficiencies to further identify the causal gene within this candidate region. Six independent RILs with the allele for relatively high fecundity and six RILs with the allele for relatively low fecundity were crossed to each of seven deficiencies that partially overlap across the QTL region (Figure 1B). From offspring we measured age specific fecundity to determine complementation. Deficiencies that do not complement the high/low fecundity alleles uncover a genomic subregion that contains a potentially causative locus for the variance in fecundity, assuming the two RIL alleles act in a semi-dominant manner and there is minimal epistasis. To statistically evaluate complementation with a likelihood-ratio test we tested the *a priori* contrast that fecundity among genotypes satisfied (A/balancer) = (B/balancer) = (A/deficiency)>(B/deficiency), where *A* is a second chromosome from high fecundity RIL allele, *B* is a second chromosome from the low fecundity RIL allele, *balancer* is the ‘wild-type’ balancer chromosome, and *deficiency* is particular aberration on the second chromosome. This contrast was only satisfied for two deficiencies: *Df(2R)enA* and *Df(2R)ED2230* (Figure 1D–1E, Figure S2A–S2E, Table S6). Importantly these deficiencies overlap only in a small region, and thus imply that the causal locus is likely to be one of the five genes within this segment: *CG7759, CG30026, CG34054, CG7763* and *Drip* (Figure 1B).

**Figure 2:**
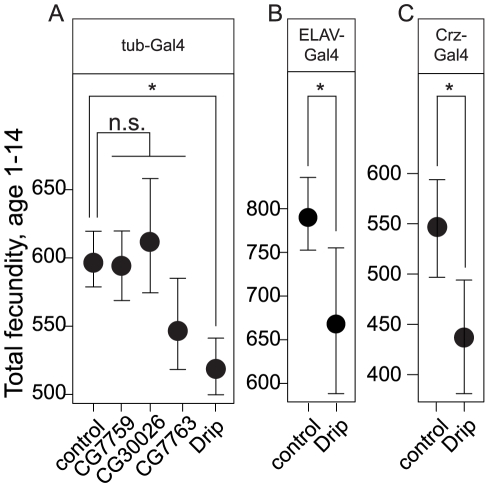
Effects of overexpression of RNAi constructs against positional candidate genes on female fecundity. (A) Ubiquitous overexpression by the *tub-Gal4* of RNAi constructs against driver for four of the five candidate genes. (B) Overexpression of Drip-RNAi with the pan-neuronal driver, *Elav*-Gal4, reduces female fecundity. (C) Overexpression of Drip-RNAi in corazonin producing neurons reduces female fecundity. Error bars represent 95% CI based on non-parametric bootstrap resampling (5000 replicates), conditional on fly. Asterisks represent significant difference from the control at *p*<0.05.

To determine which of these candidate genes could affect the observed variation in fecundity, we ubiquitously expressed RNAi from available UAS-lines for four of these five genes with the *tubulin*-Gal4 driver and measured fecundity. This experiment was not performed for *CG34054* because the appropriate RNAi line did not exist. Each of these RNAi transgenes reduced mRNA of their targeted genes by 2.5 to 3.25 log_2_-fold (all *p*<0.05, Figure S3A). Expression of *Drip*-RNAi, but not of any other candidate, significantly reduced fecundity relative to the control at *p*<0.05 (Figure 2A, Table S7).

**Figure 3:**
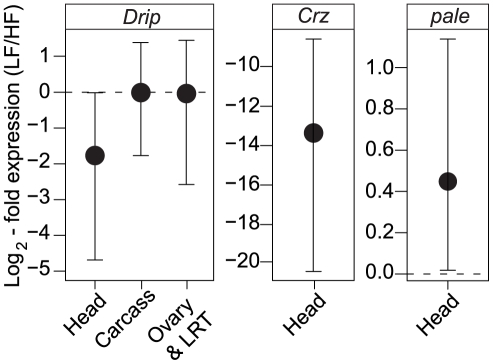
Tissue-specific expression of *Drip*, *Crz*, and *pale* between the different RIL alleles. The *y*-axis represents fold change in *Drip* expression between low- and high-fecundity alleles, normalized to differences in *Rpl32*. Error bars represent 95% CI based on permutations; see text for details. The horizontal, dashed line represent the null hypothesis of no change in gene expression.

Given that experimental repression of *Drip* reduces fecundity, we determined whether the endogenous expression of *Drip* differs amongst the high and low fecundity RIL alleles. *Drip* mRNA was measured from heads, ovaries plus lower reproductive tract (LRT) and carcasses (thorax, legs, wings, and the abdomen except the ovaries-LRT) from 3–5 day old mated females. *Drip* mRNA abundance did not differ among alleles in samples from the ovary-LRT or the carcass (ovary+LRT *p* = 0.3, carcass *p* = 0.28; Figure 3A). On the other hand, *Drip* mRNA was reduced ∼2.7 log_2_-fold in head tissue of RILs for the low fecundity allele relative to those of the high fecundity allele (*p* = 0.017, Figure 3A).

To understand how variation in Drip might contribute to differences in fecundity we sequenced the *Drip* exons in the high and low fecundity RILs. Amongst the two alleles, we observed no non-synonymous SNPs and only two synonymous SNPs (Genbank accessions JN791442-3). Natural variation in fecundity caused by variation at the Drip locus is not likely to be caused by protein variation at Drip. As alternative explanations, nucleotide polymorphisms responsible for these expression differences might be caused by the synonymous SNPs if they affect mRNA stability or processing, or by polymorphisms in 3′ or 5′ UTRs or in intronic or 5′ enhancer regions. Based on the deficiency mapping, if the causative polymorphism resides in the 5′ enhancer region, it must occur within 6.5 Kb of the transcriptional start site.

**Figure 4:**
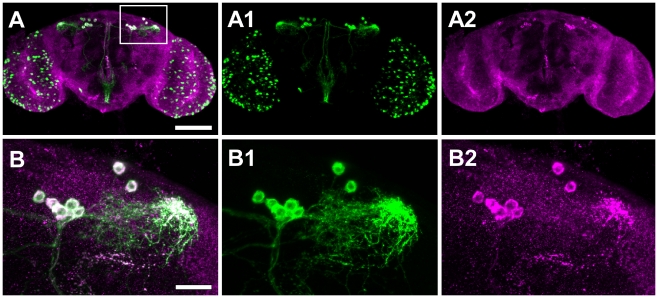
Drip is expressed in a neuropeptide corazonin (*Crz*) neurons located in protocerebrum of the brain. (A) Confocal sections of the brain of *Crz-GL4/UAS-mCD8-EGFP* female stained with anti-GFP (green, A1) and anti-Drip (magenta; A2). White indicates the overlap of these colors. Images are oriented with dorsal up. Scale bar, 100 µm. (B) Higher magnification views of the dorsolateral protocerebrum of the brain (inset box of A). Scale bar, 20 µm.

To date, Drosophila *Drip* has been reported as expressed in the malphigian tubules [39]; a gut associated organ with kidney-like functions. In contrast, we observed *Drip* expression from the adult head. We used immunohistochemistry to determine which head tissues are responsible for this expression. Drip protein is stained in about 12 neurons located in the dorsolateral posterior compartments of the protocerebrum (Figure 4A–4B′). UAS-*Drip*-*IR* (*Drip*-RNAi) expressed with the pan neuronal driver *ELAV-Gal4* effectively reduced *Drip* mRNA measured from adult heads by ∼1.75 log_2_-fold (*p*<0.001) and reduced fecundity by ∼20% (Figure 2B, Figure S3B, Table S7). Thus, neuronal expression of *Drip* is sufficient to affect fecundity.

To determine which dorsolateral posterior neurons express Drip, we screened neuropeptide-specific *GAL4* lines for co-staining with anti-Drip antibody. Drip expression coincides with *Crz*-*Gal4*, which specifically marks neurons producing the neuropeptide corazonin ([42]; Figure 4A1–4B2). *Drip*-RNAi expressed in *Crz* neurons by *Crz-Gal4* reduced both mRNA (*p* = 0.054, Figure S3C) and protein levels of Drip (Figure S4A–S4B′), and reduced fecundity by ∼20% (*p* = 8.5e-10, Figure 2C, Table S7).

Corazonin is a GnRH-like [43], stress response hormone [44] found in most insects [45]. In *D. melanogaster*, genetic ablation of corazonin producing neurons increases tolerance to starvation, osmotic and oxidative stress, decreases levels of trehalose [46], and increases triglyceride and dopamine [47] concentrations in the haemolymph. Accordingly, we tested if low and high fecundity RILs vary in *Crz* expression and if they vary in expression of the *D. melanogaster* homologue of tyrosine-hydroxylase, *pale*, the rate limiting step in the production of dopamine [48]. Low fecundity RILs show a 12 log_2_-fold decrease in *Crz* expression relative to high fecundity RILs (*p*<1e-6, Figure 3B) and a 0.5 log_2_ increase in *pale* expression (*p* = 0.01, Figure 3C). These results are consistent with the observation that exogenous application of dopamine in Drosophilid flies leads to a decrease in female fecundity [49].

Taken together, our data suggest that *Drip* mediates fecundity by altering corazonin and dopamine production. In drosophilids, genetic ablation of corazonin producing neurons increases stress resistance presumably by decreasing corazonin titer [44], [47]. Further, dopamine titer is positively correlated with stress resistance [47] and locomotor activity [50]. Here, we show that decreased *Crz* and increased *pale* expression are associated with a decrease in fecundity. Thus, these two neurohormones may contribute to the physiological basis for tradeoffs between reproduction and aspects of somatic maintenance contributing to increased stress resistance and activity. We hypothesize that Drip, an aquaporin involved in transport of small hydrophilic molecules such as water and glycerol across the plasma membrane [39], acts upstream of these neurohormones where it may function to signal hydration, nutritional status or osmolarity. If true, our model suggests that natural polymorphisms in *Drip* that affect female fecundity, a core life history trait, could be subject to balancing selection because they modulate the physiological basis for environmentally dependent life-history trade offs.

## Materials and Methods

### Fly stocks and husbandry

Initial QTL mapping was performed using twelve RILs (lines 69, 72, 94, 169, 218, 219, 252, 262, 285, 347, 369, 496). These lines are a subset of a larger population of RILs [38]. These RILs were made by crossing two isofemales lines derived from wild flies collected at the Wolfskill Orchard in Winters, CA (38°N, 121°W) in the summer of 2001. Any alleles segregating within this mapping population represent naturally segregating genetic variation. RILs were genotyped by oligoligation assay [51] at 102 SNPs along the X, 2nd and 3rd chromosomes and by PCR and Sanger sequencing at eleven additional genes near the QTL LOD peak in the RILs used in this study (see Text S1).

For phenotypic assays, RILs were reared at controlled density of 50 eggs/vial in media containing either 0.2% and 0.6% yeast by volume (YBV). Sugar, cornmeal, agar, and tegosept concentrations (11%, 8%, 5%, and 1% by volume, respectively) were kept constant. Rearing vials were kept at 25°C, 12L:12D at 40% relative humidity (RH). Each RIL was reared in four to five replicate vials for each of two replicate blocks. We also reared subsets of these RILs in 0.6% YBV under controlled densities for mRNA extraction. Extractions were made from three to five day old, mated females.

Quantitative complementation using deficiencies was performed by crossing RILs used for QTL mapping to one of seven deficiency stocks (*Df(2R)BSC328*, *Df(2R)enA*, *Df(2R)ED2155*, *Df(2R)ED2230* [courtesy of H.A.J. Müller, [52]], *Df(2R)BSC160*, *Df(2R)en30* and *Df(2R)BSC39*). Progeny of these crosses were reared at controlled density of 50 eggs/vial in media containing 0.6% YBV in four to five replicate vials.

RNAi over expression was performed using UAS-IR lines (*CG7759*, *P{KK100412}; CG30026*, *P{KK107569}*; *Drip, P{KK107343}; CG7763, P{KK105972}*) from the *phiC31* insertion collection ([53]; courtesy VDRC, Vienna, Austria). As a control, we used *y, w[1118]; P{attp, y[+], w[3′]}* which contains the *phiC31* landing site but no inverted repeat construct. These four UAS-IR lines and the control were crossed to *tubulin-Gal4* (y^1^w^*^; *P{w^+mC^, tub-Gal4}LL7/TM3, Sb^−^*). UAS-IR against *Drip* along with the control were crossed to *ELAV-Gal4* (w^*^, P{w^+mW.hs = GawB^}Elav^C155^) and *Crz*-*Gal4* [[54]; (*yw; P{w^+mC^, Crz-Gal4}*)]. Progeny of all crosses were reared in media containing 0.6% YBV as above.

### Fecundity assays

Upon eclosion, virgin females were collected over ice and placed with two OreR males in vials containing media with 2.0% YBV (all other ingredients same as above) dyed green with food coloring (McCormick & Co, Inc.) and sprinkled with live yeast granules. Flies were kept at 25°C, 12L:12D and 40% RH during fecundity assays. Vials were changed daily for 14 days and stored at 4°C until the eggs were counted.

### Ovariole number and thorax length

See Text S1.

### Variance components, genetic and phenotypic correlation calculations of recombinant inbred lines

See Text S1.

### Mapping

For QTL mapping, quantitative complementation and RNAi screens fecundity was analyzed as a function valued trait (e.g. [55]). We used a modified version of the triangular fecundity function [56] which we subsequently linearized (see SI) to facilitate efficient statistical analysis. The modified function takes the form 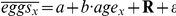, where 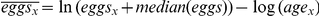, and where *eggs_x_* is the fecundity of an individual female at age *x* (*age_x_*), **R** is the set of random effects (e.g., vial effects) for a particular cross and ε is the normally distributed error. To calculate estimates of total fecundity (age 1–14) for a particular genotype we fit this function using the mixed effect model package *lme4* [57] implemented in R 2.10 [58]. We then took the integral of the fitted function from days 1–14 and back transformed 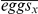 to produce estimates of total fecundity.

QTL mapping was performed with estimates of total fecundity for each RIL (see Text S1 for details) using multiple imputation [41] with 50 imputations per marker/pseudomarker and a pseudomarker step size of 3cM. We tested for association between genomic location and fecundity by fitting the full model, *y* = M_i_+E+M_i:_E+ε, and the reduced model, *y* = E+ε, where *y* is estimated fecundity, M_i_ is the effect of marker or pseudomarker *i*, E the effect of larval environment and M_i:_E their interaction. The difference in LOD score between the full and reduced model represents the strength of association between a particular genomic region and variation in fecundity segregating amongst the RILs. Statistical significance of QTL was determined by permutation testing [59].

We tested for failure to complement in quantitative complementation tests by assessing the *a priori* contrast that A/Balancer = B/Balancer = A/Deficiency>B/Deficiency where A is the high fecundity RIL allele and B is the low fecundity RIL allele. We tested this contrast by fitting mixed effect model, 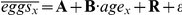 using the R package *lme4* [57], where **A** and **B** represent the fixed effect contrast matrices corresponding to [0,0,0,1] = [A/Bal, B/Bal, A/Def, B/Def] and **R** is the set of random effects that include RIL and rearing vial nested within RIL (see Text S1). In order to assess statistical significance of failure to complement we performed likelihood ratio tests between the above model and two reduced models that sequentially remove terms **B** and **A**. Two times the difference in log-likelihoods between the more complex and less complex model follows a χ^2^ distribution. Likelihood ratio tests for fixed effects such as these can be anticonservative when testing against a χ^2^ distribution with degrees of freedom equal to the difference number of parameters between the competing models (in this case, one; [60]). Therefore, we tested the likelihood ratio statistic against a χ^2^ distribution with two degrees of freedom which produces a conservative test. If the likelihood of models for a particular deficiency are significantly improved by including either **A** or **B** and if the parameter estimate for either **A** or **B** is below zero we regard that deficiency as failing to complement due to allelism.

We analyzed RNAi crosses in a similar fashion. For each Gal4 driver and UAS-RNAi cross, we fit the model 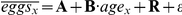 where **A** and **B** are the contrast matrices [0,1] = [control, RNAi]. As above, we tested this model against two models that sequentially remove terms **B** and **A** and compared the likelihoods of these models. If the likelihoods of models for a particular Gal4-RNAi cross are significantly improved by including either **A** or **B** and if the parameter estimate for these contrast matrices is below zero, we conclude that the particular RNAi line reduces fecundity.

### Quantitative PCR (qPCR)

mRNA transcript levels were measured with reverse-transcription qPCR. Flies were reared as above and frozen at −80°C until RNA extraction. To measure *Drip* expression in the head, ovary plus LRT and carcass in the RILs, flies were rinsed briefly in 95% ethanol, washed two times in phosphate buffered saline (PBS) and dissected in RNA Later (Qiagen). Tissue was stored overnight at 4°C in RNA Later prior to RNA extraction. RNA from the RNAi over expression experiments was extracted from whole flies for *tub*-Gal4 crosses and from heads in *ELAV*-Gal4 and *Crz*-Gal4 crosses in three biological replicates, each containing tissue from 10–15 female flies using TRIzol reagent (Invitrogen). Purity and quantity of RNA was determined spectrophotometrically (NanoDrop, ND-1000) and treated with DNase to remove residual DNA contamination (Ambion). Reverse transcription and quantification were performed using *iScript* One-Step RT-PCR kit with SYBR Green (Bio Rad) or SensiFAST SYBR One-Step Kit (Bioline) and measured on either a ABI prism 7300 Sequence Detection System (Applied Biosystems) or Eco (Illumina) qPCR machine. Gene expression for each biological replicate was measured three times (technical replicates). mRNA levels of *Rpl32* were used to normalize mRNA levels of target genes. qPCR data were analyzed using the *qpcR* package [61] in R 2.10 [58]. Reported *p* values and confidence intervals were calculated from permutation tests. For more information on primers see Text S1.

### Immunohistochemistry

3–5 day-old virgin females were dissected under PBS (pH 7.4). The brain tissues were fixed for 30 minutes at room temperature in 4% paraformaldehyde in PBS, and then were incubated in primary antibody for 48 hours at 4°C, and in secondary antibody for 24 hours at 4°C. Antibodies used were: rabbit anti-Drip ([62], 1∶500), mouse anti-GFP (1∶1000; Sigma G6539), Alexa 488 anti-rabbit (1∶1000, Invitrogen A11008), Alexa 488 anti-mouse (1∶1000, Invitrogen A11001), Alexa 568 anti-mouse (1∶1000, Invitrogen A11004). Images were acquired with a Zeiss LSM 700 and were processed in Image J [63].

## Supporting Information

Figure S1 Phenotypic and genetic correlations between fecundity and ovariole number, thorax length or development time. In (A–C), points represent total fecundity and either ovariole number (A), thorax length (B) or development time (C) of individual flies. In (D–F) points represent genetic estimates of fecundity and genetic means of ovariole number (D), thorax length (E) or development time (F). In (C), points are jittered for visual clarity. Black circles represent flies reared in media containing 0.2% yeast by volume (YBV) and white circles represent flies reared in media containing 0.6% YBV.
(PDF)

Figure S2 Results of complementation tests with deficiencies that complement the two RIL alleles. In each panel, the *x*-axis represents the tester, “wild-type” chromosome or the deficiency chromosome. The *y*-axis represents estimated fecundity. Black and white circles represent the high and low fecundity RIL alleles, respectively. Error bars represent 95% CI based on non-parametric bootstrap resampling (5000 replicates), conditional on fly.
(PDF)

Figure S3 Efficient knockdown of target genes with RNAi. (A) Log_2_ fold change in gene expression of target genes due to over expression of RNAi constructs with *tub*-Gal4 relative to the control cross, normalized to *Rpl32*. (B–C) Log_2_ fold change in *Drip* mRNA relative to control cross, normalized to *Rpl32*, caused by overexpression of RNAi construct with (B) *Elav*-Gal4 and (C) *crz*-Gal4 Error bars represent 95% CI based on permutations; see text for details.
(PDF)

Figure S4 Reduction of Drip immunoreactivity in *Crz* neurons expressing *Drip-RNAi*. The brain of *Crz-Gal4/+* (A) and *Crz-Gal4/UAS-Drip-IR* (B, B′) stained with anti-Drip antibody. (B′) Higher contrast view of (B) reveals weakly stained neurons (arrows). *Crz-Gal4* carries *UAS-Dcr-2* transgene. Scale bar, 20 µm.
(TIF)

Table S1 Mixed effect model results for ovariole number.
(DOC)

Table S2 Mixed effect model results for thorax length.
(DOC)

Table S3 Mixed effect model results for development time.
(DOC)

Table S4 Mixed effect model results for fecundity.
(DOC)

Table S5 Phenotypic and genetic correlation matrices.
(DOC)

Table S6 Quantitative complementation results.
(DOC)

Table S7 RNAi over expression results.
(DOC)

Text S1 Supplemental materials and methods.
(DOC)
