# Supplementary figures and images for: Fine-Scale Mapping of Natural Variation in Fly Fecundity Identifies Neuronal Domain of Expression and Function of an Aquaporin
Source: PLoS Genet. 2012 Apr 5;8(4):e1002631. doi: 10.1371/journal.pgen.1002631 (PMC3320613; doi:10.1371/journal.pgen.1002631)

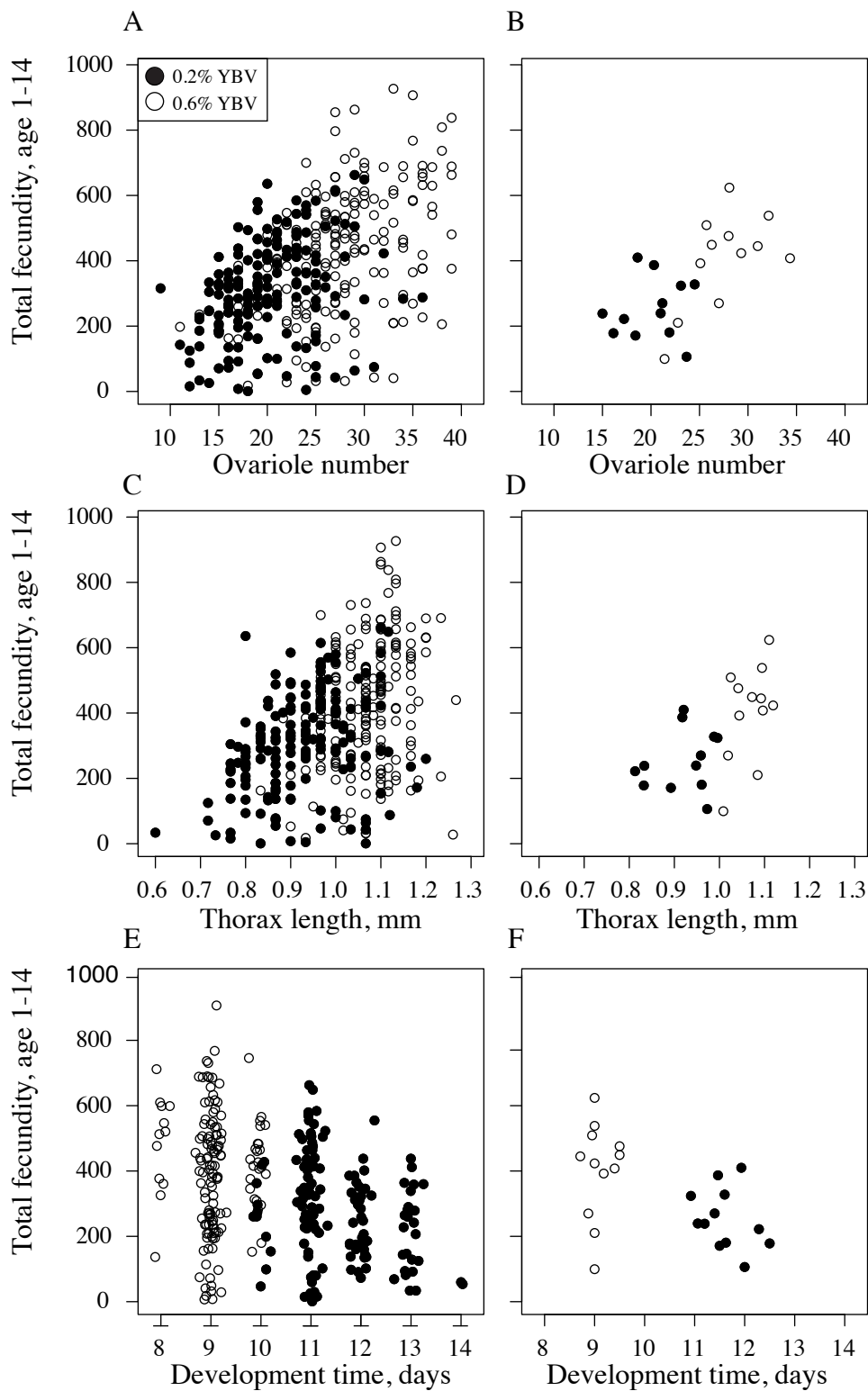

Supplement: Figure S1 — Phenotypic and genetic correlations between fecundity and ovariole number, thorax length or development time. In (A–C), points represent total fecundity and either ovariole number (A), thorax length (B) or development time (C) of individual flies. In (D–F) points represent genetic estimates of fecundity and genetic means of ovariole number (D), thorax length (E) or development time (F). In (C), points are jittered for visual clarity. Black circles represent flies reared in media containing 0.2% yeast by volume (YBV) and white circles represent flies reared in media containing 0.6% YBV. (PDF) [file pgen.1002631.s001.pdf]

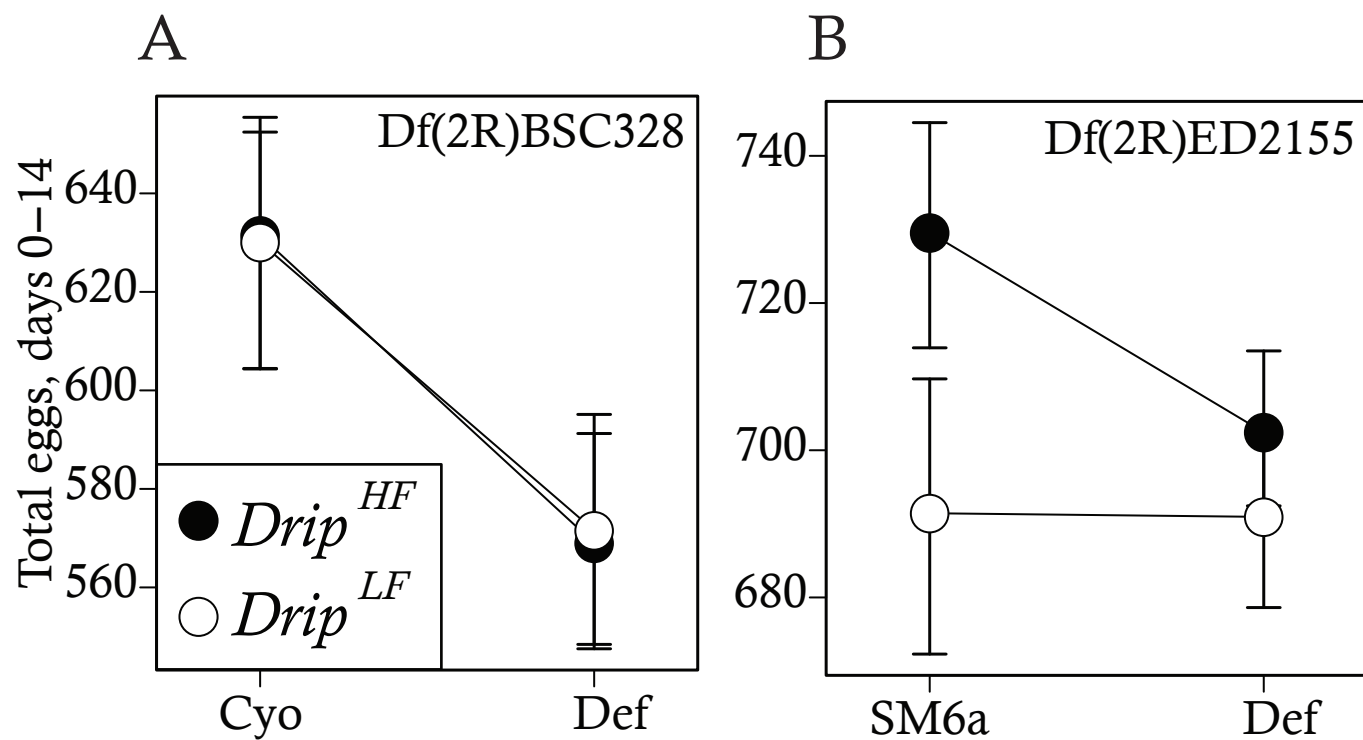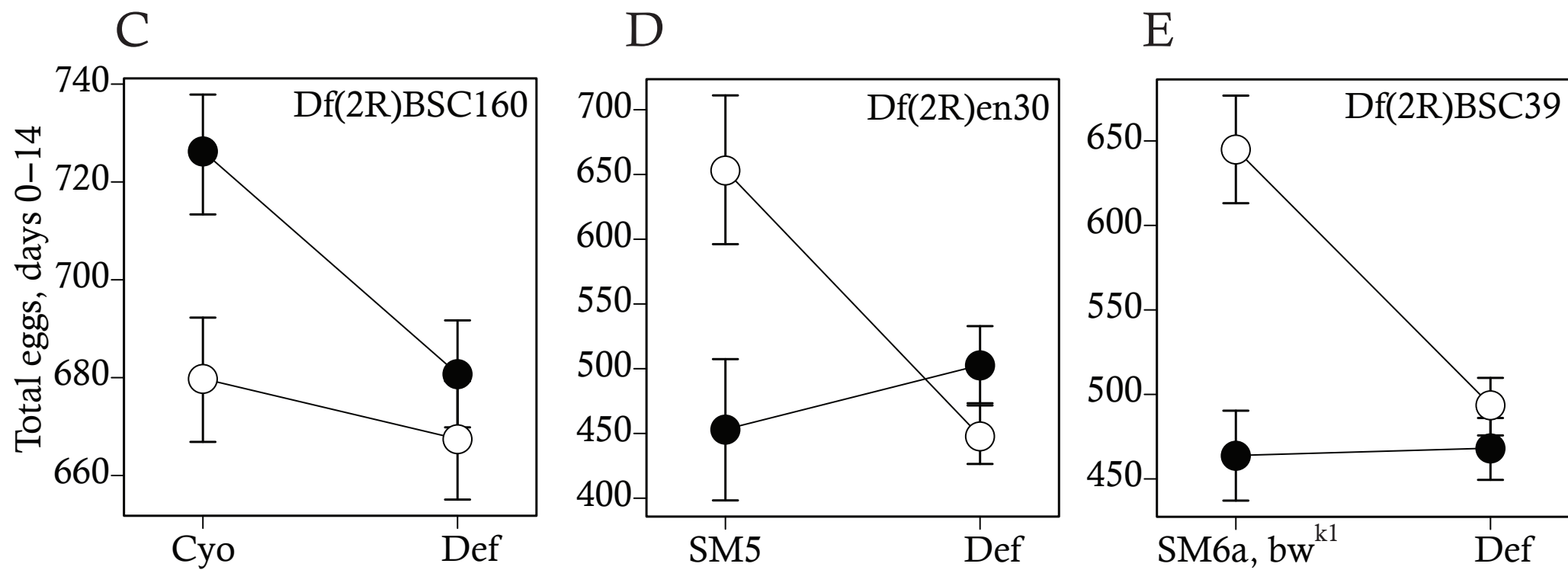

Supplement: Figure S2 — Results of complementation tests with deficiencies that complement the two RIL alleles. In each panel, the x-axis represents the tester, “wild-type” chromosome or the deficiency chromosome. The y-axis represents estimated fecundity. Black and white circles represent the high and low fecundity RIL alleles, respectively. Error bars represent 95% CI based on non-parametric bootstrap resampling (5000 replicates), conditional on fly. (PDF) [file pgen.1002631.s002.pdf]

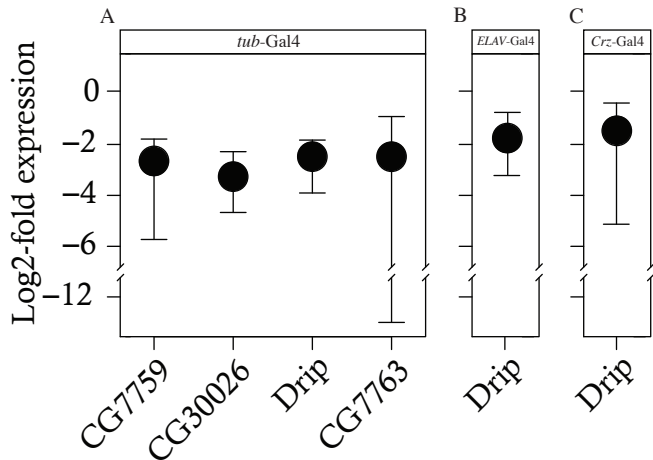

Supplement: Figure S3 — Efficient knockdown of target genes with RNAi. (A) Log2 fold change in gene expression of target genes due to over expression of RNAi constructs with tub-Gal4 relative to the control cross, normalized to Rpl32. (B–C) Log2 fold change in Drip mRNA relative to control cross, normalized to Rpl32, caused by overexpression of RNAi construct with (B) Elav-Gal4 and (C) crz-Gal4 Error bars represent 95% CI based on permutations; see text for details. (PDF) [file pgen.1002631.s003.pdf]

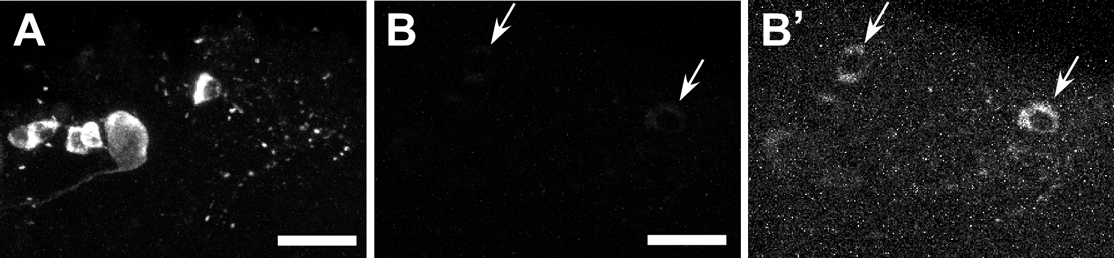

Supplement: Figure S4 — Reduction of Drip immunoreactivity in Crz neurons expressing Drip-RNAi. The brain of Crz-Gal4/+ (A) and Crz-Gal4/UAS-Drip-IR (B, B′) stained with anti-Drip antibody. (B′) Higher contrast view of (B) reveals weakly stained neurons (arrows). Crz-Gal4 carries UAS-Dcr-2 transgene. Scale bar, 20 µm. (TIF) [file pgen.1002631.s004.tif]
