# Supplementary material for: Fine-Scale Mapping of Natural Variation in Fly Fecundity Identifies Neuronal Domain of Expression and Function of an Aquaporin
Source: PLoS Genet. 2012 Apr 5;8(4):e1002631. doi: 10.1371/journal.pgen.1002631 (PMC3320613; doi:10.1371/journal.pgen.1002631)
Supplement: Table S1 — Mixed effect model results for ovariole number. (DOC) [file pgen.1002631.s005.doc]

Supplementary table 1: Mixed-effect model results for ovariole number

| Term | MS | VC | χ^2^ | df | *p-*value |
| --- | --- | --- | --- | --- | --- |
| Food | 542.25 |  | 118.01 | 1 | 1.72x10^-27^ |
| RIL |  | 2.34 | 9.70 | 1 | 0.00184 |
| Food:RIL |  | 17.57 | 22.26 | 2 | 1.46x10^-5^ |
| Block |  | 0.37 | 0.58 | 1 | 0.44 |
| Error |  | 21.16 |  |  |  |
