# Supplementary material for: Fine-Scale Mapping of Natural Variation in Fly Fecundity Identifies Neuronal Domain of Expression and Function of an Aquaporin
Source: PLoS Genet. 2012 Apr 5;8(4):e1002631. doi: 10.1371/journal.pgen.1002631 (PMC3320613; doi:10.1371/journal.pgen.1002631)
Supplement: Table S2 — Mixed effect model results for thorax length. (DOC) [file pgen.1002631.s006.doc]

Supplemental table 2: Mixed-effect model results for thorax length

| Term | MS | VC | χ^2^ | df | *p-*value |
| --- | --- | --- | --- | --- | --- |
| Food | 0.2957 |  | 133.61 | 1 | 6.65x10^-31^ |
| RIL |  | 3.76x10^-4^ | 6.13 | 1 | 0.013 |
| Food:RIL |  | 4.16x10^-3^ | 12.07 | 2 | 0.0023 |
| Block |  | 6.71x10^-5^ | 0.46 | 1 | 0.50 |
| Error |  | 7.95x10^-3^ |  |  |  |
