# Supplementary material for: Fine-Scale Mapping of Natural Variation in Fly Fecundity Identifies Neuronal Domain of Expression and Function of an Aquaporin
Source: PLoS Genet. 2012 Apr 5;8(4):e1002631. doi: 10.1371/journal.pgen.1002631 (PMC3320613; doi:10.1371/journal.pgen.1002631)
Supplement: Table S3 — Mixed effect model results for development time. (DOC) [file pgen.1002631.s007.doc]

Supplemental table 3: Mixed-effect model results for development time

| Term | MS | VC | χ^2^ | df | *p-*value |
| --- | --- | --- | --- | --- | --- |
| Food | 173 |  | 455.71 | 1 | 4.11x10^-101^ |
| RIL |  | 0.094 | 42.17 | 1 | 8.37x10^-11^ |
| Food:RIL |  | 0.11 | 12.07 | 2 | 0.000141 |
| Block |  | 0.00 | 0.00 | 1 | 0.00 |
| Error |  | 0.42 |  |  |  |
