# Supplementary material for: Fine-Scale Mapping of Natural Variation in Fly Fecundity Identifies Neuronal Domain of Expression and Function of an Aquaporin
Source: PLoS Genet. 2012 Apr 5;8(4):e1002631. doi: 10.1371/journal.pgen.1002631 (PMC3320613; doi:10.1371/journal.pgen.1002631)
Supplement: Table S4 — Mixed effect model results for fecundity. (DOC) [file pgen.1002631.s008.doc]

Supplemental table 4: Mixed-effect model results for fecundity

| Term | MS | VC | χ^2^ | df | *p-*value |
| --- | --- | --- | --- | --- | --- |
| Food | 2.11 |  | 447 | 1 | 2.46x10^-99^ |
| Age | 276.16 |  | 10613 | 1 | 0 |
| RIL |  | 0.0080 | 1108 | 1 | 5.18x10^-243^ |
| Age:RIL |  | 0.00018 | 165 | 3 | 1.2x10^-35^ |
| Food:RIL |  | 0.148 | 160 | 3 | 1.33x10^-34^ |
| Age:Food:RIL |  | 0.00023 | 49 | 4 | 5.44x10^-10^ |
| Block |  | 0.0012 | 22 | 1 | 2.36x10^-6^ |
| Error |  | 0.08817 |  |  |  |
