# Supplementary material for: Fine-Scale Mapping of Natural Variation in Fly Fecundity Identifies Neuronal Domain of Expression and Function of an Aquaporin
Source: PLoS Genet. 2012 Apr 5;8(4):e1002631. doi: 10.1371/journal.pgen.1002631 (PMC3320613; doi:10.1371/journal.pgen.1002631)
Supplement: Table S5 — Phenotypic and genetic correlation matrices. (DOC) [file pgen.1002631.s009.doc]

**Supplementary table 5: Phenotypic and genetic correlation matrices**

|  |  | **P^a^** | | | |  | **G^b^** | | | |
| --- | --- | --- | --- | --- | --- | --- | --- | --- | --- | --- |
|  |  | Ovariole number | Thorax length | Devel. time | Fecundity |  | Ovariole number | Thorax length | Devel. time | Fecundity |
| **Simple** ^c^ | Ovariole number | - | **0.80**  (0.72 –  0.86) | **-0.34**  (-0.51 –  -0.12) | **0.36**  (0.15 –  0.48) |  | -0.42  (-0.8 –  0.20) | **0.96**  (0.86 – 0.99) | -0.36  (-0.77 – 0.27) | 0.04  (-0.54 – 0.60) |
|  | Thorax length | **0.58**  (0.45 – 0.69) | - | **-0.41**  (-0.57 –  -0.21) | **0.35**  (0.17 – 0.50) |  | 0.60  (0.04 – 0.87) | -0.18  (-0.68 – 0.43) | -0.49  (-0.83 – 0.18) | 0.19  (-0.43 – 0.67) |
|  | Devel. time | 0.06  (-0.17 –  0.29) | 0.12  (-0.11 –  0.34) | - | **-0.26**  (-0.43 –  -0.08) |  | 0.20  (-0.42 – 0.69) | 0.16  (-0.46 –  0.67) | 0.35  (-0.28 – 0.77) | -0.10  (-0.63 –  0.51) |
|  | Fecundity | **0.45**  (0.29 – 0.58) | **0.30**  (0.13 – 0.45) | -0.10  (-0.27 – 0.07) | - |  | 0.58  (0.01 – 0.86) | 0.48  (-0.17 – 0.81) | 0.14  (-0.47 – 0.66) | 0.56  (-0.02 – 0.86) |
|  |  |  |  |  |  |  |  |  |  |  |
| **Partial** ^d^ | Ovariole number | - | **0.78**  (0.65 – 0.91) | 0.05  (-0.13 – 0.24) | -0.02  (-0.28 – 0.25) |  | - | **0.96**  (0.88-0.99) | 0.37  (-0.63 – 0.90) | -0.21  (-0.89 -0.75) |
|  | Thorax length | **0.69**  (0.57-0.82) | - | -0.23  (-0.42 -0.037) | 0.29  (0.09 – 0.49) |  | 0.49  (-0.19 – 0.88) | - | -0.44  (-0.9 – 0.4) | 0.21  (-0.75 – 0.875) |
|  | Devel. time | 0.09 (-0.2 – 0.38) | -0.01  (-0.29 – 0.27) | - | -0.11  (-0.36 -0.12) |  | 0.16  (-0.62 – 0.8) | 0.04  (-0.57 – 0.65) | - | -0.21  (-0.88 – 0.71) |
|  | Fecundity | 0.20  (-0.09 – 0.5) | 0.05  (-0.24 – 0.35) | -0.22  (-0.47 – 0.038) | - |  | 0.38  (-0.53 – 0.92) | 0.21  (-0.46 – 0.80) | -0.18 (-0.79 – 0.55) | - |

^a^ Phenotypic correlation matrices based on phenotypes from 236 and 208 individuals from the 0.2% and 0.6% yeast by volume treatments, respectively.

^b^ Genetic correlation matrices based on line means of 12 RILs.

^c^ Values above the diagonal in each submatrix (**P** or **G**) represent Pearson correlation coefficients between traits within the 0.2% yeast by volume treatment, values below the diagonal represent correlation coefficients between traits within the 0.6% yeast by volume treatment. Values along the diagonal in the **G** matrix represent correlations within traits between environments. Underlined values represent correlations significantly different from zero at a nominal *p<*0.05, underlined values in bold represent significant correlations after Bonferroni correction. Values in parentheses represent 95% confidence intervals for correlation coefficients.

**^b^** Values above the diagonal in each submatrix (**P** or **G**) represent partial correlation coefficients between traits within the 0.2% yeast by volume treatment, values below the diagonal represent partial correlation coefficients between traits within the 0.6% yeast by volume treatment. Underline and bold indicate significance at *p* < 0.05 before and after Bonferroni correction, respectively. Values in parentheses are confidence intervals based on 5000 bootstrap simulations.
