# Supplementary material for: Fine-Scale Mapping of Natural Variation in Fly Fecundity Identifies Neuronal Domain of Expression and Function of an Aquaporin
Source: PLoS Genet. 2012 Apr 5;8(4):e1002631. doi: 10.1371/journal.pgen.1002631 (PMC3320613; doi:10.1371/journal.pgen.1002631)
Supplement: Table S6 — Quantitative complementation results. (DOC) [file pgen.1002631.s010.doc]

Supplementary table 6. Quantitative complementation results.

| Deficiency | **A**^1^ | $c_{A}^{2}$^2^ | **B** |  |
| --- | --- | --- | --- | --- |
| *Df(2R)BSC328* | 0.40 | 0.49 (0.78) | -0.044 | 0.87 (0.64) |
| *Df(2R)ED2155* | -0.0043 | 0.11 (0.94) | 0.001 | 0.44 (0.81) |
| ***Df(2R)enA*** | **0.0087** | **15.01 (0.00055)** | **-0.0079** | **13.13 (0.0014)** |
| ***Df(2R)ED2230*** | **-0.0030** | **7.82 (0.020)** | -0.0031 | 3.05 (0.22) |
| *Df(2R)BSC160* | 0.0032 | 3.88 (0.14) | -0.0027 | 3.46 (0.17) |
| *Df(2R)en30* | 0.12 | 0.53 (0.76) | -0.018 | 3.81 (0.15) |
| *Df(2R)BSC39* | 0.022 | 0.80 (0.67) | 0.0023 | 0.76 (0.96) |

^1^ Values represent maximum likelihood estimates of contrast effect.

^2^ Values represent χ^2^ statistic for contrast effect and values in parentheses represent *p*-values for these statistics tested against a χ^2^ distribution with two degrees of freedom.
