# Supplementary material for: Fine-Scale Mapping of Natural Variation in Fly Fecundity Identifies Neuronal Domain of Expression and Function of an Aquaporin
Source: PLoS Genet. 2012 Apr 5;8(4):e1002631. doi: 10.1371/journal.pgen.1002631 (PMC3320613; doi:10.1371/journal.pgen.1002631)
Supplement: Table S7 — RNAi over expression results. (DOC) [file pgen.1002631.s011.doc]

Supplementary table 7. RNAi over expression

| Gal4 driver | RNAi line | **A**^1^ | $c_{A}^{2}$^2^ | **B** |  |
| --- | --- | --- | --- | --- | --- |
| *tub-*Gal4 | *CG7759* | -0.048 | 0.26 (0.88) | 0.0061 | 4.52 (0.10) |
|  | *CG30026* | -0.012 | 0.19 (0.91) | 0.0032 | 1.11 (0.57) |
|  | ***Drip*** | **-0.077** | **14.30 (0.00078)** | 0.0016 | 0.37 (0.83) |
|  | *CG7763* | -0.043 | 4.86 (0.088) | 0.00012 | 0.0016 (0.99) |
| *ELAV-gal4* | ***Drip*** | 0.0033 | 0.098 (0.63) | **-0.0012** | **8.06 (0.018)** |
| *crz-gal4* | ***Drip*** | **-0.13** | **41.73 (8.5e-10)** | 0.0025 | 0.22 (0.895) |

^1^ Values represent maximum likelihood estimates of contrast effect.

^2^ Values represent χ^2^ statistic for contrast effect and values in parentheses represent *p*-values for these statistics tested against a χ^2^ distribution with two degrees of freedom.
