# Supplementary material for: Fine-Scale Mapping of Natural Variation in Fly Fecundity Identifies Neuronal Domain of Expression and Function of an Aquaporin
Source: PLoS Genet. 2012 Apr 5;8(4):e1002631. doi: 10.1371/journal.pgen.1002631 (PMC3320613; doi:10.1371/journal.pgen.1002631)
Supplement: Text S1 — Supplemental materials and methods. (DOC) [file pgen.1002631.s012.doc]

**Text S1.**

**Derivation of fecundity function.** The classic triangular fecundity function [1] is a four parameter, non-linear model that captures the typical fecundity profile of *D. melanogaster*. It takes the form,

,

where *eggs_x_* is the daily fecundity at *age_x_.* The estimated parameters, *m*, *a, age_0_* and *b* most strongly reflect maximum egg production, the rate of increase in egg production prior to maximum fecundity, age at first reproduction and the rate of decrease in egg production after maximum fecundity, respectively. We simplified this function in two ways. First, we removed *age*_0_ because in our experiments*,* egg production begins at age 1. Second, we simplified the term to *age_x_* because for *D. melanogaster,* egg production increases more or less linearly up to peak reproduction. This simplified, two-parameter model thus takes the form

.

We linearized this model by taking the logarithm of both sides and subtracting the offset term, log(*age_x_*), from the dependent variable which produces the model,

.

This model is computationally efficient and stable compared to the four or two parameter non-linear models above, particularly for mixed models that incorporate random effects such as rearing vial.

**Morphological measurements**. Ovariole number and thorax length were measured as in Bergland *et al*. [2]. Briefly, thorax length was measured as the longest distance from the tip of scutellum to the most anterior part of the thorax with an occular micrometer accurate to 1/30mm. Ovariole number was measured in both ovaries. Development time was scored for the second block only.

**Fine scale mapping of QTL***.* We refined the breakpoints of the QTL that affects fecundity for the 12 lines used in the original QTL mapping between the two markers (*lola* and *synaptogyrin)* that flank the QTL peak. We sequenced intronic and coding sequences from six genes 5’ (*luna, CG9084, CP47e, dare, shavenoid, sprite*) and five genes 3’ (*Sobp, CG13188, pds5, jelly belly, CG33964)* to the original LOD peak in the 12 RILs where there was either missing genotype data at *lola* or *synaptogyrin* or where there was a recombination breakpoint between either *lola* or *synaptogyrin* and the marker most associated with fecundity, *Drip*. Primers and PCR conditions available upon request. Primers were designed using Primer3 [3] using the *D. melanogaster* assembly 5.0 genome sequence (http://flybase.org). PCR reaction mixtures contained 10 parts PCR buffer (Denville), 10 parts 10 mM dNTPs, 2 parts 10μM primer (each), 2.5 parts genomic DNA and 72.5 parts H_2_O. PCR reactions were performed on Eppendorph or MJ thermocyclers using the following program: 95° for 5 minutes, 35 cycles of 95° 30”, 55° 30”, 72° 1’30”, followed by a final extension of 72° for 10 minutes. PCR products were cleaned for sequencing using a GeneClean II kit (QBioGene) or by the sequencing facility. Sequencing was performed by Cogenics or by the University of Rhode Island Genomics (Kingston, RI) center with an ABI 3700 sequencer. DNA sequences were aligned with Sequencher 4.8 (Gene Codes), CLUSTAL ([4]) or MUSCLE ([5]) implemented in eBioX.

**Variance components and correlations of RILs**. In order to determine the extent of genetic, environment and genotype-environment variation for ovariole number, thorax length, development time and fecundity within the mapping population, we used mixed effects models implemented in *lme4* [6] using maximum likelihood. For ovariole number, thorax length and development time, we fit the model , where *y* is one of the three phenotypes listed above, *food* is the fixed effect of larval diet (0.2% or 0.6% yeast by volume), *RIL* the random effect of recombinant inbred line, their interaction, and *block* the random effect of rearing block. For fecundity, we fit the model, , *age_x_* is the fixed effect of age, and,, are interaction terms interactions. In these analysis, we were interested in the statistical significance of each model term and thus we tested the statistical significance of each term by calculating models without that particular term and performing likelihood ratio tests. Degrees of freedom are calculated as the difference in parameter number between models which, for random effects, also includes covariance terms.

We calculated phenotypic and genetic correlations between traits within environments and tested if each correlation was significantly different from zero. For simple, Pearson correlation coefficients, confidence intervals and hypothesis tests for each correlation were calculated using the *cor.test* function in *R* 2.10 [7]. For partial correlations, we used the *corpcor* function in R and generated confidence intervals and p-values through 5000 bootstrap simulations. We corrected for multiple testing by applying a Bonferroni correction for phenotypic and genetic correlations separately.

**qPCR conditions and primers**. qPCR primers were designed from the *D. melanogaster* genome assembly 5 (http://flybase.org) using Primer3 [3]. Primers were designed to span introns which should minimize spurious results due to accidental DNA contamination. qPCR reaction mixtures and amplification protocols followed manufacturer recommendations. We measured the disassociation curves to ensure primer dimer did not affect the outcome. *Drip* transcript was measured using the forward primer (FP) 5'-ACC ACA AAT CGC ATT CAC CT -3' and reverse primer (RP) 5'-ACC AAC GAT CAG GAA TCC AA-3'; *CG7759* with FP 5'-CAC CAT TGA TGA GGA GCT GA-3' and RP 5'-AAG AAG TTG GAT GGC TGA CG-3'; *CG30026* with FP 5'-GCA ATC CGG CAG TAA AAT GT-3' and RP 5'-GTA GCA GCA GCG GTA ACA AA-3'; *CG7763* with FP 5'-GAC ACA CGG TAC AGC AAT GG-3' and RP 5'-CAA AGC ATC GTG CCA GTT TA-3'; *pale,* FP 5’-CAT GCC AAT CTG GAG AAC CT, RP 5’-GAC TCC GAC TGC TCC TGT TC-3’; *crz,* with FP 5’-GGG ACT CAC GGA TCT CTA CG-3’ and RP 5’-TTT CTG GCA ATC AGT GAA CG-3’*.* The control gene, *Rpl32* was measured with FP 5'-AAT GAT GTG CGA GTG CCG AG-3' and RP 5'-CAA TGG TGC TGC TAT CCC AAT C-3'.

**Supplemental references**

1. McMillan I, Fitz-Earle M, Robson DS (1970) Quantitative genetics of fertility. I. Lifetime egg production of Drosophila melanogaster--theoretical. Genetics 65: 349-353.

2. Bergland AO, Genissel A, Nuzhdin SV, Tatar M (2008) Quantitative trait loci affecting phenotypic plasticity and the allometric relationship of ovariole number and thorax length in Drosophila melanogaster. Genetics 180: 567-582.

3. Rozen S, Skaletsky H (2000) Primer3 on the WWW for general users and for biologist programmers. Methods in molecular biology 132: 365-386.

4. Thompson JD, Higgins DG, Gibson TJ (1994) CLUSTAL W: improving the sensitivity of progressive multiple sequence alignment through sequence weighting, position-specific gap penalties and weight matrix choice. Nucleic Acids Research 22: 4673-4680.

5. Edgar RC (2004) MUSCLE: multiple sequence alignment with high accuracy and high throughput. Nucleic Acids Research 32: 1792-1797.

6. Bates D, Maechler M (2009) lme4: Linear mixed-effects models using S4 classes. . R package version: 0.999375-32 ed.

7. Team RCD (2009) R: A language and environment for statistical computing. Vienna, Austria: R Foundation for Statistical Computing.
